# Developmental regulation of nucleoplasmin function by phosphorylation, glutamylation, and methylation

**DOI:** 10.1186/1756-8935-6-S1-P61

**Published:** 2013-03-18

**Authors:** Takashi Onikubo, Joshua J Nicklav, Wei-Lin Wang, Jeffrey Shabanowitz, Donald F Hunt, David Shechter

**Affiliations:** 1 Department of Biochemistry, Albert Einstein College of Medicine, Yeshiva University, Bronx, NY, USA; 2 Department of Chemistry, University of Virginia, Charlottesville, VA, USA

## 

Nucleoplasms (Npm) is an abundant storage chaperone for histones H2A and H2B in the oocyte, egg and early embryo of *Xenopus laevis.* Regulation of Nucleoplasmin histone binding and release is critical to its developmental function in establishing zygotic chromatin. To test the function of Nucleoplasmin post-translational modifications in regulation of its function, we determined its full complement of PTMs, characterized their dynamic changes during development, and tested their role in modulating plasmid supercoiling. Here we present the full mass spectrometric analysis of nucleoplasmin from two oogenic states: the non-activated, oocyte form, and the activated, egg form. We characterize the changes in phosphorylation that occur upon oocyte maturation and also show that nucleoplasms is differentially polyglutamylated between the two states. Specifically, we show an increase in phosphorylation of nucleoplasms from a majority of two within oocytes to a majority of six within eggs. This hyperphosphorylation occurs upon germinal vesicle breakdown induced by progesterone treatment. We demonstrate dynamic post-translational addition of up to five glutamyl groups (glutamylation) to residues within the second acidic stretch of nucleoplasms. Glutamylation is an isopeptide addition of a glutamic acid to the γ-carboxyl of a primary chain glutamate residue. We also identify a highly-abundant, C-terminal arginine methylation on arginine 187 within the “GRGR” motif that we previously demonstrated to be catalyzed by the arginine methyltransferase complex PRMT5-MEP50. Npm hyperphosphorylation is present from egg-laying until the mid-blastula transition, concomitant with the period of transcriptional repression, while arginine methylation and glutamylation persist through gastrulation. Finally, we demonstrate that Nucleoplasms phosphorylation negatively regulates its ability to promote supercoiling, indicative of altered histone release. We conclude that developmentally dynamic changes in Nucleoplasms phosphorylation modulate its histone binding and release.

